# Artificial intelligence and Big Data in neurology

**DOI:** 10.1590/0004-282X-ANP-2022-S139

**Published:** 2022-08-12

**Authors:** Edson Amaro

**Affiliations:** 1 Hospital Israelita Albert Einstein, Big Data, São Paulo SP, Brazil.; 2 Universidade de São Paulo, Faculdade de Medicina, Instituto de Radiologia, São Paulo SP, Brazil.

**Keywords:** Big Data, Artificial Intelligence, Neurology, Big Data, Inteligência Artificial, Neurologia

## Abstract

Recent advances in technology have allowed us access to a multitude of datasets pertaining to various dimensions in neurology. Together with the enormous opportunities, we also face challenges related to data quality, ethics and intrinsic difficulties related to the application of data science in healthcare. In this article we will describe the main advances in the field of artificial intelligence and Big Data applied to neurology with a focus on neurosciences based on medical images. Real-World Data (RWD) and analytics related to large volumes of information will be described as well as some of the most relevant scientific initiatives at the time of this writing.

## RESUMO

Os recentes avanços na tecnologia nos permitiram acessar uma infinidade de conjuntos de dados pertencentes a várias dimensões da neurologia. Juntamente com as enormes oportunidades, também enfrentamos desafios relacionados à qualidade dos dados, ética e dificuldades intrínsecas relacionadas à aplicação da ciência de dados na área da saúde. Neste artigo descreveremos os principais avanços no campo da inteligência artificial e Big Data aplicados à neurologia com foco nas neurociências baseadas em imagens médicas. Dados do mundo real (RWD) e análises relacionadas ao grande volume de informações serão descritos, bem como algumas das iniciativas científicas mais relevantes no momento da redação deste artigo.

## INTRODUCTION

### What is Big Data and why do we need artificial intelligence

Technological advances in medicine have been evolving gradually over the past three decades. However, in the past 10 years we have seen an exponential increase in the number of publications related to data analytics of large samples of patients as well as the use of neural networks to analyze complex data sets^1^. In fact, by the end of 2020 it was estimated that we should have reserved around 5,200 Gb per individual in our population - on average (considering that our society is characterized by inequalities, data per capita also varies a lot)^2^.

**Figure 1.**
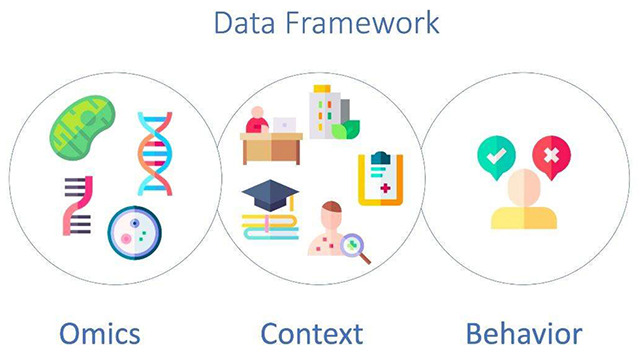
Conceptual Data Frame for organizing Datasets in health. The three human-centric dimensions involve biological data (“Omics”), daily-living context (“Ambient”) and decision-making (“behavior”).

Before we begin to describe the main advances in this field it is important to define a few aspects related to the definition of terms. In this paper we should use the term Big Data Analytics as the new class of strategy and tools designed to analyze large volumes of complex data. Sejdic and Falk have defined Big Data in the context of Health care as, “…high volume, high diversity biological, clinical, environmental, and lifestyle information collected from single individuals to large cohorts, in relation to their health and wellness status, at one or several time points”^3^. And we should understand that this definition includes the sense of usefulness in different contexts, aiming at application in single cases. Paradoxically, we need to be able to understand human variability through Big Data analytics as a critical step to provide insights required in Precision Medicine to address a single patient. In order to reach this goal, we propose a human-centric conceptual framework converging Behavior, Biological and Ambient data (Figure 1). The unimaginable complexity of analytics of such complex and large datasets can only be faced using sophisticated computer systems^1^. In this scenario the use of advanced analytical tools, most of them heavily based in Artificial Intelligence (AI) solutions, is paramount in dealing with such large data sets^4^.

## ARTIFICIAL INTELLIGENCE, MACHINE LEARNING AND DEEP LEARNING

The AI field is not new: the first concepts date from as early as the 1940s^5^. Nowadays - and especially over the recent years, AI has taken the main stage in various dimensions of our daily life. Although this long delay can be explained by many factors, reduced computational processing cost and increased data access were the two key points allowing AI to flourish. There are many definitions for AI and one of the simplest is: a computational system aimed at imitating human intelligence. It can be divided into two main types: a) specific, ‘weak’, or narrow AI (NAI) representing solutions dedicated to solve single, focused problems (however usually very complex or tedious for humans) and b) full, or global, ‘strong’ AI - also called Artificial General Intelligence (A.G.I.) representing a concept more linked to what we define as general intelligence: the ability to understand or learn any intellectual task that a human being can. To the date of this writing, no consensus has been achieved regarding the existence of systems capable of A.G.I., although some authors have pointed out that complex neural network designs have come close to this definition. By and large, the narrow AI is the most common form of AI used in daily routine - and has revolutionized several areas in our society: from entertainment to financial. Interesting to note is that ‘narrow’ AI is responsible for the ‘largest range’ of applications based on Big Data. Another term frequently found when dealing with sophisticated algorithms is machine learning (M.L.). In fact, M.L. is one type of artificial intelligence, based on computational structures designed to^6^ resemble neural networks. The way M.L. works is based on designing computer codes in a way that it can “learn” by comparing its results with reality. In this way, M.L. can be trained to perform as accurately as possible depending on data quality. A more sophisticated type of M.L. is called Deep Learning (D.L.), which basically represents a very sophisticated ML architecture. For instance, a recent D.L. network called GPT3 uses more than 185 billion parameters and is capable of reading more than 500 billion *tokens* (a concept that can be thought of as an approximation of a word). Astonishingly, GPT3 has been able to compose poems in various author styles and have outperformed other networks in language translation tasks^6^.

**Figure 2.**
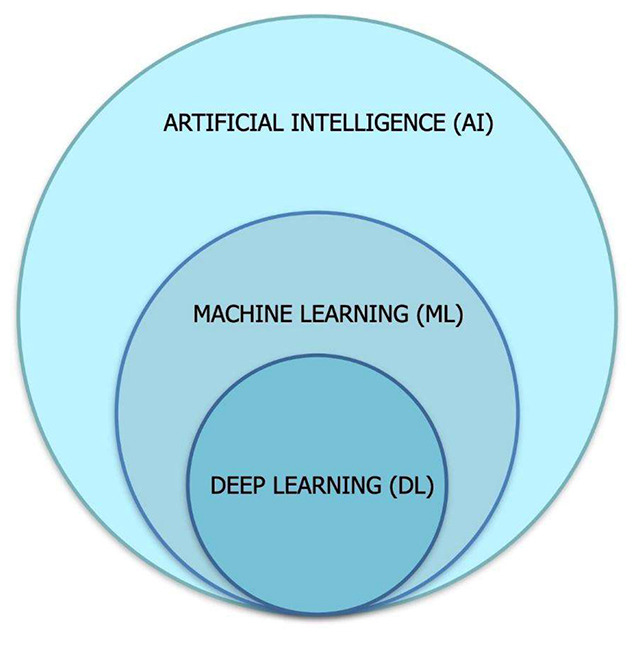
Differences and relationship between Artificial Intelligence, Machine Learning and Deep Learning.

These terms can be confusing, but a conventional way to express the relationship between AI, M.L. and D.L. is the following: AI is the broader term, applicable to a technique that allows computers to mimic human intelligence, using logic, if-then rules, decision trees, and M.L. (including D.L.); M.L. is the AI ​​subset, which includes more sophisticated statistical techniques that allow machines to improve tasks with experience. The category includes deep learning; finally, D.L. is the subset of M.L. comprising algorithms that allow the software to train itself to perform tasks such as speech and image recognition, resulting from multilayered neural networks for large amounts of data (Figure 2). 

Taken together, AI benefits from a large volume of data and Big Data Analytics heavily depends on AI techniques. It would not be possible to enable the generation of new insights and decision-rich information without both Big Data and AI combined.

This challenge cannot be tackled solely by technical feats from the AI methods alone: it requires an interdisciplinary team effort. The combination of *networks* formed by human and artificial intelligence is perhaps the most challenging and key ingredient^7^ ^,^ ^8^. 

## THE USE OF BIG DATA IN NEUROLOGY

A few examples illustrate the use of AI studies in neurology. One example of how methods based on artificial intelligence can help to better understand diagnostic information present in magnetic resonance images is provided by a study performed in a sample of elderly patients with Alzheimer’s disease compared to a healthy age-matched control group^9^. Since the key sign of A.D. in MRI data is volume loss and considering that healthy aging is also related to brain volume loss, it is even more difficult to distinguish late-onset A.D. patients from controls. In that particular study it was possible to differentiate between the two groups by using solely the information contained in the structural MRI data by using a support factor machines algorithm which achieved a discriminating power of 88.2% [CI95% ; 72.5%-96.7%] (Sensitivity=92.8% [CI 95% ; 66.1%-99.8%], Specificity=85.0% [CI 95% ; 62.1%- 96.8%].

Another challenge to applying artificial intelligence algorithms to medical data relies on a subgroup of patients that are not easily found within one single context. This is the case of rare disease patients or patients in an ICU sector of a hospital. In both cases it is difficult to concentrate on a large number of patients in the same condition in just one institution. First, when one aims at analyzing a large number of patients one must certainly deal with different protocols, cultures and access to treatment or diagnostic criteria. These challenges of multi- center studies make it even more complex to analyze the data. In particular, the use of AI algorithms based on D.L. techniques require a large number of samples. Moreover, it is key for clinical applications to validate their results in different contexts in order to better understand the performance regarding false positives or false negatives. Successful external validation methods are fundamental for clinical adoption of AI. As an example, our group has provided insight on how to validate artificial intelligence algorithms in different hospitals in low/middle income countries^7^.

An interesting use of AI is to help bridge the gap between pathology and radiology. Algorithms based on D.L. can be used to better assess the spatial correspondence between pixels from pathology tissue samples and MRI data. For instance, Ushizima et al. have developed a computational pipeline to identify and segment imunostained phospho-tau antibodies areas in billion-pixel digital pathology images and successfully process over 500 slides from two whole human brains spanning several terabytes of images. The proposed convolutional neural network for immunohistochemistry samples, IHCNet, was able to match the tau-marked regions to MRI brains providing a pathway to better understand the role of in vivo neuroimaging techniques^10^.

Several other possibilities using AI applied to neurology, especially neuroimaging, have emerged: from emergency or in hospital use^11^ to large scale studies^12^. However, there are still challenges to be tackled. A particular point is related to ethical and responsible use of Big Data and AI technologies. As we have learned from past experience, there is a need to carefully assess the possibilities of health gain and examine possible legal consequences. Recent studies have shown previously unforeseen possibilities, such as decoding brain states or even visual information^13^ and their corresponding ability to expose personal experiences of previously inaccessible information^14^. A recommended reading is an interesting historical view with future perspectives provided by Christos Davatzikos^15^.

## BIG DATA NEUROIMAGING

Neuroimaging and graphic methods are a valuable source of information. There is a variety of neuroimaging data types and hybrid equipment (i.e. PET-MR systems; EEG-MR/fNIRS-EEG and other combinations allow for simultaneous data acquisition) that are able to provide converging information in a one-stop-shop manner. These datasets can provide information not only related to brain form and function in healthy subjects, but also in patients with injury and dysfunctional conditions. 

When analyzing data from multiple sources, commonalities present in brain injury and/or disease mechanisms can be extracted from large-scale multimodal neuroimaging. Using sophisticated image data analytics, it is possible to provide evidence that indicates possible novel biomarkers to further explore in normative reference samples. Moreover, some bias from ‘conventional’ science may be tested with large datasets. For instance, one study using functional Magnetic Resonance Imaging showed that, after analyzing 3,317 subjects, the results from a previously considered ‘large sample’ of 272 subjects were, actually, not reproduced^16^.

Currently there are various initiatives based on patient populations which have considerable potential for revealing disease mechanisms combined with genetic, phenomic, and other associated data sources in a Big Data environment^17^. A remarkable achievement was a publication of 123,984 MRI scans to depict biomarkers throughout human lifespan between 115 days post-conception to 100 years of age^18^. In the following paragraphs we summarize some of the OpenScience Large Dataset Initiatives available for improving knowledge particularly in Neurology (among other medical specialties):

- *The Human Connectomme Project* is a multicenter initiative funded by the US government comprising more than 1,200 subjects that were analyzed by high resolution MRI techniques and specific demographic and behavior data^19^. The project has evolved into a Connectome Coordination Facility that added some groups of patients covering different contexts: normal lifespan, young adults and connectomes related to specific diseases (Epilepsy Connectome Project; Anxiety and Depression in Teenagers; The Structural and Functional Connectome Across Alzheimer's Disease Subtypes and Human Connectomes for Low Vision, Blindness, Sight Restoration). A large number of publications have followed that initiative - details can be found at https://www.humanconnectome.org/;
- *The Alzheimer’s Disease Neuroimaging Initiative (ADNI)* is one of the first Dataset initiatives comprising not only MRI data but also clinical information about each subject including recruitment, demographics, physical examinations, and cognitive assessment data as well as PET data (PIB, FDG, FLORBETAPIR, FLORBETABEN, and TAU IMAGING)^20^. The initiative has also added 818 Whole Genome Sequences (WGS) from its participants (128 with AD, 415 with MCI, 267 controls and 8 of uncertain diagnosis) in 2012. To date the ADNI dataset has been used in more than 3,300 scientific publications (https://adni.loni.usc.edu/);
- *The United Kingdom BioBank (UKBB)* is a large-scale biomedical database and research resource, containing genetic and health information from 500,000+ UK participants aged between 40 and 69 recruited since 2006 and living in the UK, as part of a large-scale prospective study. The database contains high-resolution Brain MRI as well as blood, urine and saliva samples, together with detailed information about their lifestyle and clinical visits. The study uses three 3T MRI systems dedicated to collect neuroimaging data, and in 2017 the initiative released a paper analyzing functional and structural brain MRI from 15,847 individuals, all collected under the same imaging protocol - an important difference from the ENIGMA consortium (see below). The data is made widely accessible by UK Biobank to researchers around the world. In particular, recent advances in polygenic risk scores for cardiac disease have been possible at least in part due to UKBB data^21^. A cardiovascular investigation of 100,000 individuals from the UKBB has been announced, comprising brain, cardiac and abdominal MRI, carotid ultrasound and DEXA^22^ (https://www.ukbiobank.ac.uk/);
- *Cambridge Centre for Ageing and Neuroscience (Cam-CAN),* a large-scale collaborative research project at the University of Cambridge, UK together with the European Union Horizon 2020 LifeBrain project^18^ ^,^ ^23^ ^,^ ^24^. The Cam-CAN project uses epidemiological, cognitive, and neuroimaging data to understand how individuals can best retain cognitive abilities into old age (https://www.cam-can.org/);
- *The Adolescent Brain Cognitive Development (ABCD)* Study is a Research Consortium^24^ involving 21 research sites in the USA recruiting 11,880 children with ages ranging from 9-10. Participants will be evaluated for neurocognition, physical and mental health, social and emotional functions, and culture and environment. The study collects structural and functional brain imaging, bioassays, genetic and epigenetic data (https://abcdstudy.org/about/);
- *Cuban Human Brain Mapping Project (CHBMP)* is a multimodal neuroimaging and cognitive dataset from 282 young and middle-aged healthy individuals acquired from 2004 to 2008 as a subset of a larger sample of 2,019 participants^25^. Data contains resting-state electroencephalograms (EEG), magnetic resonance images (MRI), psychological tests and demographic information (age, gender, education, ethnicity, handedness, and weight) (https://chbmp-open.loris.ca/).

## CHALLENGES IN GENETICS BIG DATA ANALYTICS AND AI

Due to a large variability and complexity of data, added to the fact that it is virtually impossible to anonymize the data (bringing challenges for privacy data protection) the use of genetics in Big Data is still an untamed frontier. Even though initiatives have demonstrated the potential of Big Data Analytics applied to large genetic datasets, there are a few concerns. One of the first initiatives to use Big Data capabilities to better understand Human Genetics in neurology was the Enhancing NeuroImaging Genetics through Meta-Analysis (ENIGMA) Consortium. The initiative was formed in 2009 and is based on analyzing results (not raw data) from various researchers - typically aiming at tens of thousands of individuals in order to understand the effect of genetic variants in brain phenotype. The format is based on a meta-analytical platform to perform statistically sound analyses. It has more than 2,000 participating scientists from 45 countries, and over 50 working groups mainly organized toward producing disease-oriented research (substance use disorders, schizophrenia, bipolar disorders, major depression, posttraumatic stress disorder, obsessive compulsive disorder, epilepsy, and stroke)^26^. More information can be found at https://enigma.ini.usc.edu/.

Recently Naslavsky et al.^27^ have published a large data set of WGS data comprising 1,171 elderly subjects from a cohort based in São Paulo, Brazil. They were able to detect more than 76 million variants of which 2 million were not yet described in previously published WGS data sets. Moreover, this population sample has been studied regarding various aspects related to their characteristics from literacy, past clinical history and behavioral measures. Brain images from the same sample were acquired using a 3T MR system and an initial whole-brain quantitative analysis have replicated age-related changes and shown interesting features relative to male/female intracranial CSF spaces across several decades^28^. We hope the similar initiatives can promote and further develop the use of Big Data Analytics in our population.

## WHERE TO START: SHARING INFORMATION AND EXPERIENCES

Innovative solutions are increasingly part of neurology, and in Medicine in general. However, is it understandable that we take a few cautionary steps in order to adopt technical solutions concerning patient diagnosis, treatment and prognosis. Continuous medical education courses are gradually adding Data Science in their curricula. The approaches towards adoption of AI and Big Data in daily practice are part of a much greater endeavor: it involves digital literacy. Emphasizing responsible use of these technologies, its drawbacks and opportunities should be emphasized. We should not aim to transform physicians into data scientists. Rather, organizing interdisciplinary and friendly environments can be very productive. These experiences should be guided by adaptive strategies to bridge the gap between healthcare professionals and mathematicians and computer scientists. Datathon - a joint word resulting from adding “data” + “hackathon” - is an interesting approach. It is based on forming interdisciplinary teams, accentuating application of the hackathon model to data analytics and provides an effective method to ‘break the ice’ between individuals with different backgrounds. The experience is not rarely associated with intensive exchange of ideas and frequently results in scientific production, strengthening teamwork and building the framework for new projects^29^ ^,^ ^30^.
